# Zinc oxide nanoparticle-reinforced sodium alginate/hydroxyapatite scaffolds for osteoporosis treatment in fragility fracture patients: Development and characterization using artificial neural networks (ANNs) modeling

**DOI:** 10.22038/ijbms.2024.78958.17077

**Published:** 2024

**Authors:** Yuanyuan Zhou, Shujuan Wang, Yuanyuan Hu

**Affiliations:** 1 Department of Orthopedics, Nanjing Drum Tower Hospital, The Affiliated Hospital of Nanjing University Medical School, Nanjing 210008, Jiangsu, China

**Keywords:** Biodegradable scaffolds, Fragility fractures, Intervention strategy, Osteoporosis, Personalized assessment

## Abstract

**Objective(s):**

Osteoporosis is a significant public health concern due to its association with fragility fractures. Despite experiencing such fractures, many patients remain at high risk of future fractures due to inadequate management and treatment of their underlying osteoporosis. This research presents a comprehensive intervention to enhance osteoporosis management in patients with fragility fractures. The intervention involves a thorough personalized assessment of fracture risk using clinical factors and bone density testing, followed by customized treatment based on the individual’s fracture risk level. It also addresses non-compliance through patient education, counseling, reminders, and improved care coordination among acute, primary, and specialty providers.

**Materials and Methods:**

The aim is to create and characterize sodium alginate/hydroxyapatite (HA) scaffolds reinforced with zinc oxide nanoparticles (ZnO-NP) for bone tissue engineering. Freeze-drying was used to produce scaffolds with 0–15% ZnO-NP. Analyses confirmed the composite structure, uniform ZnO-NP distribution, and decreasing pore size with higher ZnO-NP content. Mechanical testing showed increased compressive strength with greater ZnO-NP.

**Results:**

The scaffolds exhibited over 70% porosity, neutral pH, and increased apatite deposition and bioactivity with higher ZnO-NP. They also demonstrated decreased swelling and strong antibacterial activity against *Escherichia coli *and *Staphylococcus aureus*, making them a promising candidate for bone regeneration.

**Conclusion:**

Additionally, the researchers used an artificial neural network (ANN) to better understand the relationships between various scaffold properties, and the ANN-based predictions showed that changes in pore size and porosity affect the other properties, with acceptable error compared to experimental results.

## Introduction

Osteoporosis is a significant public health concern due to its link with fragility fractures. Patients who have experienced a fragility fracture are at high risk of future fractures, yet their underlying osteoporosis is often inadequately managed (1-3). Common challenges include lack of comprehensive fracture risk assessment, poor treatment adherence, and insufficient coordination between acute fracture care and long-term osteoporosis management providers. Addressing these barriers is crucial to enhancing osteoporosis care for patients who have sustained fragility fractures (4, 5). Bone is one of the hardest types of connective tissue, with various functions, including protecting vital organs. Bone can produce blood cells and serves as a reservoir for essential minerals, such as calcium and phosphorus. These unique properties, including high mechanical strength and self-healing capabilities, have made bone a subject of great interest for biomedical researchers and chemists (6, 7). The mineral phase of bone, which constitutes 65–70% of its composition, is primarily hydroxyapatite (HA), a member of the calcium phosphate family with both amorphous and crystalline forms. While HA exhibits desirable characteristics like bioavailability, non-degradation, bone growth stimulation, and tissue adherence, its poor mechanical properties, and brittleness limit its use as a standalone implant material for load-bearing applications (7-9). 

To address these limitations, researchers have explored reinforcement strategies, such as incorporating secondary particles, long fibers, metal dispersions, and carbon nanotubes (9-11). The field of hard tissue engineering leverages these enhanced HA-based composites, along with living cells, to facilitate the repair of damaged tissues by mimicking the extracellular matrix. Biomaterials engineers have recognized the potential of sodium alginate for the repair of cartilaege, bone, and skin tissues. One critical component in tissue engineering and bone repair is the bone scaffold, which plays a central role in guiding bone formation. Various scaffold fabrication techniques, such as freeze-drying, 3D printing, electrospinning, and the space holder method, have been explored to produce porous materials with characteristics similar to natural bone tissue and minimal side effects (12-14). To further improve the properties of HA/Na-Alg composites, researchers have explored the incorporation of antimicrobial agents, such as silver, gold, and zinc oxide (ZnO) nanoparticles. ZnO nanoparticles have shown excellent antimicrobial effects and have been used to produce porous, biodegradable, and biocompatible bone scaffolds when combined with HA and alginate hydrogel (15-16). These advanced bone bio-nanocomposite scaffolds hold promise for the repair and regeneration of bone tissue while preventing infections (17-19). This study proposes a multi-faceted intervention to improve osteoporosis management in patients with fragility fractures. The approach involves comprehensive fracture risk assessment, patient-tailored treatment, and interventions to enhance adherence and care coordination. If effective, this coordinated, risk-stratified strategy could have significant public health impact by preventing future fractures in high-risk patients. In recent decades, humans have faced an array of bone and skin tissue damage, such as burns and fractures. While some lesions can be treated conventionally, others remain untreatable, often involving substantial tissue loss that challenges the body’s natural repair mechanisms.

## Materials and Methods

***Optimizing post-fracture osteoporosis care***

The current treatment rates are suboptimal even after fracture: Systematic reviews demonstrate only 30–60% of patients receive any osteoporosis treatment in the 12 months following a fragility fracture requiring medical care. This represents a missed opportunity to prevent recurrent fractures. Untreated or under-treated osteoporosis is a modifiable risk factor that demands attention. Many clinical guidelines recommend beginning treatment based on the presence of a single fragility fracture. However, this “one size fits all” approach ignores significant variation in an individual’s true underlying risk. Precise risk stratification could optimize the balance of benefits and harms from treatment. Non-adherence reduces the effectiveness of treatment: Poor persistence with oral bisphosphonates averages around 50% after one year of therapy. Non-adherence attenuates the fracture risk reduction afforded by these medications substantially. Behavioral and educational components are needed to boost real-world effectiveness. Fracture liaison services have demonstrated improved uptake of bone-sparing therapy and cost-effectiveness, but availability globally remains limited. Better integration is required between acute fracture care, primary care, and specialist osteoporosis services. While fracture liaison models have advanced care, greater focus on individualized risk assessment, precise treatment targeting, adherence promotion, and care coordination could drive outcomes even higher. A comprehensive, evidence-based intervention deserves thorough evaluation. In summary, contemporary approaches fail to optimize post-fracture osteoporosis management. The proposed multi-modal intervention aims to fill these gaps through precise risk assessment, tailored treatment decisions, adherence support, and coordinated care across healthcare interfaces. This includes elements not yet fully addressed in existing fracture liaison service models. A randomized trial could determine whether such a strategy leads to meaningfully improved outcomes. The present study utilized a feedforward artificial neural network (ANN) with a hidden layer to model and predict the relationships between key parameters of the scaffolds developed for bone tissue engineering applications. Specifically, ANN was employed to estimate the crystal size (in nanometers), ultimate stress (in megapascals), and antibacterial activity (as a percentage) across a broader range of pore size (in microns) and porosity percentage values investigated in the experimental component of the research. Additionally, linear regression analysis was conducted to evaluate the error of the neural network predictions in comparison to the empirical results obtained. The predictive modeling undertaken using ANN, along with the review and evaluation of the estimation process, are reported in the current study.

*Fracture risk assessment*

Following recruitment after a fragility fracture, patients would complete a standardized risk factor assessment, including age, gender, prior fracture, parental hip fracture, smoking status, glucocorticoid use, rheumatoid arthritis, and secondary causes of osteoporosis. A Dual-energy X-ray absorptiometry (DXA) bone density test of the hip and spine would then be performed to determine T-scores at these sites. FRAX or other validated models would incorporate this data to generate an individualized 10-year absolute fracture risk score for major osteoporotic and hip fractures. To optimize medication-taking behavior, patients would receive education on osteoporosis and treatment rationale via individual counseling sessions. Written materials and discussion would cover potential side effects and emphasize long-term persistence, which is necessary for fracture protection. Reminder devices (e.g., medication blister packs and smartphone apps) and follow-up phone calls would be utilized to support initial adherence. This component draws on techniques from motivational interviewing and cognitive behavioral therapy, which have been shown to be effective in promoting medication adherence in chronic illnesses.

*Care coordination*

A fracture liaison nurse coordinator or case manager would oversee each patient’s progress. This individual would facilitate handovers between acute orthopedic teams, primary care physicians, bone health specialists, and allied health professionals. Standardized documentation and communication processes (e.g., discharge summaries and management plans) would be implemented. Follow-up DXAs and risk reassessments would also be scheduled centrally to minimize loss to follow-up. The coordinator role aims to improve continuity across clinical interfaces.

*Evaluation and outcomes monitoring*

 Rates of risk assessment completion, treatment initiation, persistence, adherence, and clinical outcomes would be continuously monitored. At defined intervals, patients would undergo repeat clinical assessment, DXA testing, and fracture documentation to track changes in risk profile and fracture occurrence over time. Secondary outcomes may include healthcare utilization, costs, and quality of life.

*Pilot randomized controlled trial*

We propose evaluating this multi-modal osteoporosis intervention using a pilot randomized controlled trial. The objectives would be to establish feasibility, estimate effect sizes, and inform sample size calculations for a full-scale study.

*Clinical impact*

Fewer fragility fractures would mean less pain, disability, loss of independence, and mortality for patients who have osteoporosis. Healthcare systems could experience lower fracture-related costs through fewer hospitalizations, surgeries, nursing home admissions, and outpatient visits over time. Broader application across healthcare systems treating osteoporotic fractures could translate to substantial gains in population health. Demonstrating the benefits of a coordinated, risk-stratified approach could drive broader adoption of evidence-based osteoporosis care processes internationally. Elements like enhanced fracture risk assessment, tailored treatment decisions, adherence promotion, and care coordination could be more routinely implemented in guidelines and clinical practice. Knowledge gaps around optimizing post-fracture care could be addressed, informing future interventions aiming to maximize the real-world effectiveness of osteoporosis therapies. Establishing the efficacy and cost-effectiveness of a comprehensive strategy may support business cases for health system investment in expanded fracture liaison services globally.

*Health equity evaluation*

System-level coordination may help overcome disparities seen with fragmented care models, promoting equitable access to high-quality osteoporosis management for all socioeconomic/demographic groups. Educational components could be adapted and evaluated to meet the needs of culturally diverse patient populations. Detailed data collection on intervention processes, individual risk profiles, and longitudinal outcomes would generate new empirical evidence to advance the science of osteoporosis disease management. Novel methodologies piloted, such as combining clinical risk factors with DXA scans in tailoring treatment intensity, may stimulate fresh lines of research inquiry. Rigorously demonstrating that a multi-faceted intervention can enhance osteoporosis treatment practices and prevent recurrent fractures after initial low trauma fractures would represent an essential step toward optimizing outcomes for the many patients affected by this serious health condition worldwide. The proposed strategy and study aim to advance this vision of improving post-fracture care.

*Resource requirements*

Conducting this multi-component intervention trial across multiple sites would require substantial resources for additional personnel like case managers and coordinators, technology support, education materials, bone density testing, and data management. Securing necessary funding could prove difficult. It may be challenging to recruit a sufficient sample of eligible post-fracture patients within a reasonable time frame across participating hospitals. Retaining participants through the entire follow-up period would also require ongoing engagement efforts to minimize attrition biasing results. Standardizing complex multi-modal interventions across varied real-world clinical settings is not feasible. Ensuring the consistent delivery of core intervention components would require close monitoring to interpret outcomes meaningfully. Preventing control participants from receiving intervention elements like risk assessment or educational support delivered as usual care may be impractical depending on the settings. Contamination could dilute observed treatment effects. Findings may have limited generalizability to non-research healthcare contexts or populations if reliant on intensive research protocols instead of scalable implementations. External validity thus requires a pragmatic trial methodology. Ascertaining recurrent clinical fractures typically requires 2-5 years of follow-up, increasing study duration and costs substantially. Alternative feasible designs and outcome measures warrant consideration. Fracture type, location, and severity may differentially impact outcomes like non-union, disability, and risk of recurrence. Stratifying analyses to account for clinical heterogeneity adds complexity. Addressing challenges around resources, recruitment, intervention fidelity, generalizability, and long-term follow-up through innovative trial designs and mixed methods research will, therefore, be crucial to maximizing the internal and external validity of this evaluation. To operationalize the proposed research study effectively across multiple clinical sites, a thorough implementation plan addressing organizational, practical, and resource considerations would need to be developed. This section outlines key elements that could form part of such an implementation strategy. An oversight committee comprising representatives from stakeholder organizations would provide strategic guidance and resolve issues as they arise. A dedicated research coordinator and site coordinators would manage day-to-day implementation. Ethical approvals would be obtained from all relevant research ethics boards prior to initiation. Data sharing agreements between sites and a plan for protecting the privacy/confidentiality of patient information would also be formalized.

*Intervention delivery and standardization*

Detailed manuals of procedures define each intervention component and activities of coordinators, clinicians, and support staff. Training sessions using simulated patients ensure understanding prior to launch. Fidelity checklists allow monitoring of whether core elements (e.g., risk assessment content and reinforcement protocols) are delivered as intended at each session. Technology platforms minimize data duplication. Evaluations identify needed modifications to content, delivery, or timelines. Education documents inform control participants and clinicians about study procedures while blinding them to full intervention details. Guidelines establish parameters for usual care elements that should not be provided (e.g., bone density testing and formal osteoporosis education). Masking of group assignments may not be feasible - but study personnel responsible for assessment, data entry, and analysis will remain blinded. Standard communication records document all patient contacts by research/clinical staff. Central coordination and monitoring limit missing/implausible values. Data quality reports identify errors necessitating correction/clarification. Access is permission-based to respect patient privacy. Regular backups protect against system failures. Auditing verifies accuracy.

*Sustainability and scale-up*

If demonstrating effectiveness, strategies exploring means to continue essential services post-project require consideration. This may involve integrating roles/processes into routine clinical workflows and budgets, expanding sites through phased roll-outs, or transitioning to other long-term funding models. Piloting aspects that facilitate maintenance/growth sets the stage for real-world implementation, assuming positive results. Mixed methods evaluation assesses facilitators, barriers, and opportunities for wider scale-up. Embedding programs institutionally enhances sustainability.

*Preparation of materials*

 The present study investigated the influence of zinc oxide nanoparticles (ZnO-NP) on sodium alginate (Na-Alg) and HA. Commercially available HA with 97% purity and 40–60 nm particle size, as well as low viscosity Na-Alg (100-300cP), were utilized. The required solvents, including acetic acid and phosphate-buffered saline (PBS), were procured. Simulated body fluid (SBF) was prepared following the Kokubo method, using various salts and chemicals. Porous Na-Alg scaffolds containing antibacterial ZnO-NP were fabricated using the freeze-drying technique. Na-Alg was dissolved in hot water, and acetic acid was added to accelerate the polymerization process. Different ratios of ZnO-NP and a fixed amount of HA were then incorporated into the Na-Alg solution, which was subsequently poured into Petri dishes. The samples were frozen at -70 °C for 40 hr and then subjected to freeze-drying for 48 hr at -45 °C and 0.1 bar pressure. This resulted in the production of bio-nanocomposite scaffolds with varying amounts of ZnO-NP, while the HA and Na-Alg levels were maintained constant.

*Materials characterization*

 The phase characteristic and structure composition changes of the sample after the addition of ZnO-NP in the product were evaluated by the XRD method with a given copper lamp with a specific voltage. Using XRD patterns, the size of the powdered HA produced by the Williamson-Hall method was determined. For calculations using the Williamson-Hall method, the peaks of the crystalline plates with Miller’s indices were used. Fourier-transform infrared (FTIR) spectroscopy, using a Thermo Nicolet FTIR Nexus 670 instrument, was employed to identify the functional groups and bonds formed within the samples across the wavelength range of 400–4000 cm^-1^. To prepare the samples for FTIR analysis, a KBr primer sample was first created, with a sample-to-KBr ratio of 1:100. This mixture was then ground and pressed into a thin film using a pressure-coating apparatus before being placed on the FTIR device to examine the established bonds. The microstructure and surface morphology of the specimens were analyzed using a scanning electron microscope (SEM), specifically the model AIS2100 manufactured by SERONTECHNOLOGIES in South Korea. To prepare the samples for SEM imaging, they were coated with a thin layer of gold. The surface of the samples was then imaged using the SEM device. The pore size of the samples was subsequently measured using the ImageJ software, as described in the reference. 

*Mechanical properties evaluation*

 The samples were mechanically compressed using the Hounsfield machine. In this way, the compressive strength, elastic coefficient, and maximum strain tolerance were measured by the samples. Experiments were carried out at room temperature at a 5 mm/min speed. All samples had a width of 21 mm, a thickness of 0.07 mm, and a length of 30 mm. Liquid displacement method was used to determine the porosity percentage of scaffolds of bio-nanocomposites. The constant water content (V1) was poured into a graduated cylinder. The cylinder is used to soak the dry scaffold. The cylinder’s new volume contains water and dry V2 scaffolds. For better water penetration into the scaffold, the scaffolding was kept in water for 3 hr so no air bubbles remained. The remaining volume of water is after removing wet scaffolds from the V3 cylinder. The porosity (%) of scaffolds was calculated using the following equation (Formula 1):

Porosity (%) =) V_1_−V_3_/V_2_−V_3_(×100 (1)

*Biological properties evaluation*

 A bioactive agent should have the ability to bind to body tissues. In the case of a bone substitute, the ability to form at the level of the structure of the HA layer can be a good measure of the bioactivity of the biomaterial. In order to measure the biocompatibility of the composites, two pieces of specimens were placed in SBF in an incubator at 37 °C for 28 days. 

The Kokubo method was used to prepare the SBF solution (20-24). Potassium chloride, Calcium chloride, magnesium chloride, sodium chloride, sodium sulfate, sodium bicarbonate, dipotassium phosphate, hydrochloric acid, and Tris(hydroxymethyl) aminomethane were used for this purpose. The concentration of various ions in this solution is comparable to that of human blood plasma in Table 1. In order to evaluate the water absorption capacity of the four samples, a water absorption test was carried out for samples for 24 hr. A cubic piece of the specimen with dimensions of 1×1×2 cm was first weighed using a digital scale for the prepared swelling examination. Then, the components were placed in an incubator at 37 °C to simulate the human body environment with 15 ml of phosphate-buffered saline (PBS). Samples were taken out of the PBS solution every four hours to calculate the inflation rate. The sample change in the solution was then taken from filter paper and measured by weighing scale. Finally, the water absorption rate or inflation rate of the samples was calculated using formula 2:

Swelling % =(W_1_−W_0_)/W_0 _× 100 (2)

In this formula, W0 is the weight of the samples in dry state, and W1 is the weight of the specimens in wet state after water absorption (25, 26).

*Artificial neural networks (ANNs) modeling*

The current research employed a feedforward ANN to predict the changes in crystal size (in nanometers), ultimate stress (in MPa), and antibacterial activity (as a percentage) across a wide range of pore size (0 to 100 microns) and porosity percentage (0 to 85%) values observed in the experimental samples. The neural network architecture consisted of input nodes for pore size and porosity percentage, a hidden layer with five neurons (calculated as twice the number of inputs plus one for faster convergence), and output nodes for crystal size, ultimate stress, and antibacterial activity. A non-linear sigmoid activation function was utilized, given the non-linear nature of the relationships, which enables more accurate predictions and faster convergence of the network. The error function was optimized using the gradient descent algorithm during the training process. Additionally, the input data was normalized prior to training the network, and the final predicted results were denormalized to ensure they fell within the approved range. The accuracy of the neural network predictions was evaluated by comparing the fitted linear regression diagram to the y=x line, representing 100% accurate estimation. The error of the neural network was further quantified through linear regression analysis of the normalized predicted results.

**Table 1:** Concentration of ions in SBF: Simulated body fluid (SBF) and their comparison with the values of these ions in human blood plasma

**Ion**	**Density (mmol/dm** ^3^ **)**	
	Simulated body fluid	Human blood plasma
**Na** ^+^	142.0	142.0
**K** ^+^	5.0	5.0
**Mg** ^+2^	1.5	1.5
**Ca** ^+2^	2.5	2.5
**Cl** ^-^	147.8	103.0
**HCO**_3_^-^	4.2	27.0
**HPO**_4_^-2^	1.0	1.0
**SO**_4_^-2^	0.5	0.5

**Figure 1:**
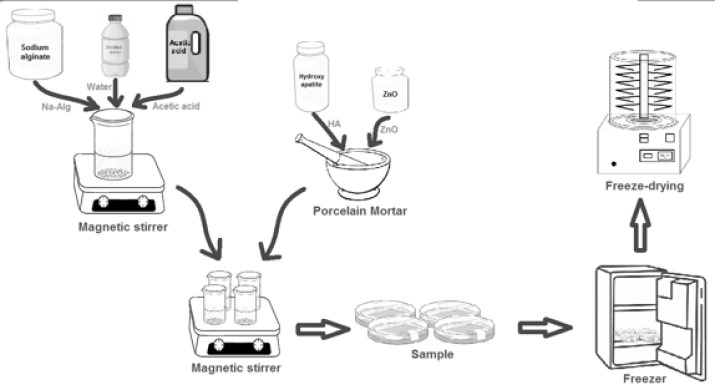
Schematic representation of the design disc bio-nanocomposite produced using antibacterial and sodium alginate with freeze-drying technology and mechanical mixing for biomedical application

The material was fabricated using freeze-drying technology and mechanical mixing techniques with the aim of developing a biomedical application. The schematic representation provides a visual overview of the key steps involved in the production of the bio-nanocomposite disc. It shows the use of freeze-drying and mechanical mixing as the main manufacturing processes, along with the incorporation of antibacterial agents and sodium alginate as the primary components of the material. The purpose of developing this bio-nanocomposite disc is indicated as a biomedical application, suggesting potential uses in the healthcare or medical field.

**Figure 2:**
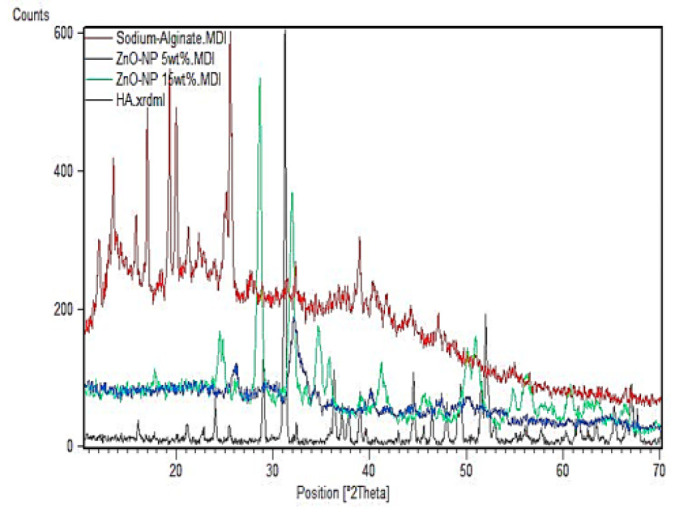
XRD patterns of the sample containing 5 wt%, 15 wt% ZnO-NP in the sodium alginate- HA

XRD: X-ray diffraction; HA: Hydroxyapatite; ZnO-NP: Zinc oxide nanoparticles

**Figure 3:**
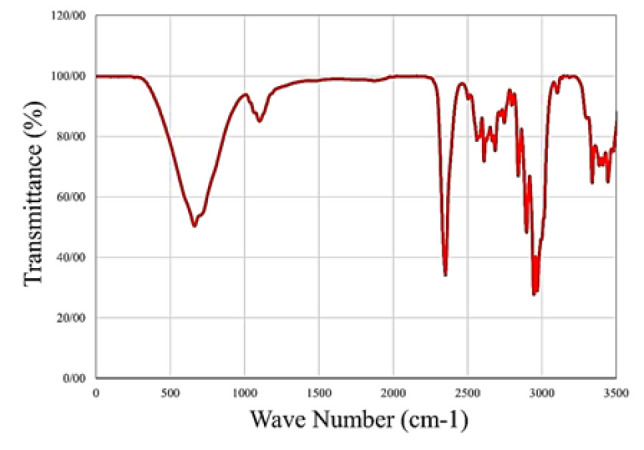
FTIR spectra of bio-nanocomposite scaffolds with varying ZnO-NP concentrations

Also, HA shows five important infra-red bands at 576 cm^-1^, 601 cm^-1^, 631 cm^-1^, 1033 cm^-1^, and 3566 cm^-1^. Among these bands, there are two bands at 3566 cm^-1 ^and 631 cm^-1^, which belong to the hydrogen bond of OH-ion in tensile and free state, respectively

FTIR: Fourier-transform infrared; ZnO-NP: Zinc oxide nanoparticles; HA: Hydroxyapatite

**Figure 4:**
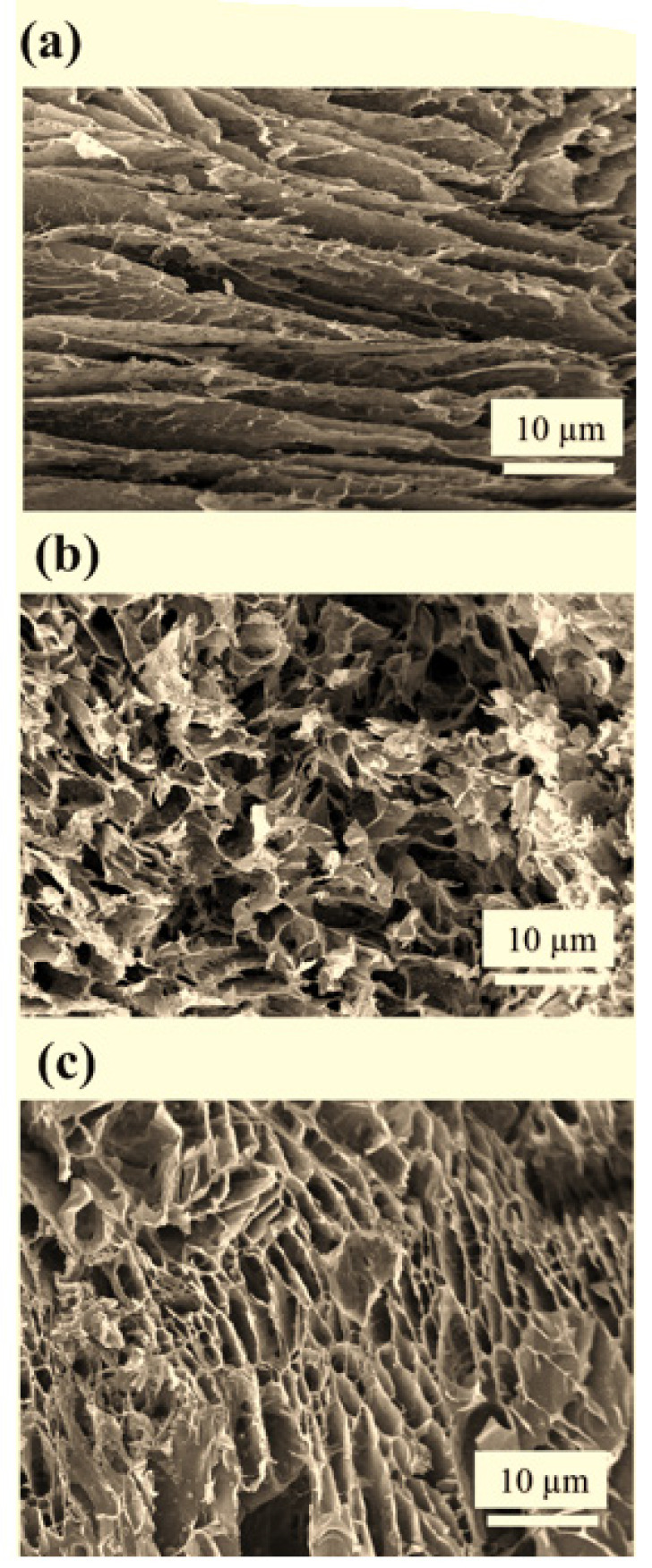
SEM images of bio-nanocomposite scaffolds containing a) 5 wt%, b) 10 wt%, and c) 15 wt% additive and bioceramic

SEM: Scanning electron microscope

**Figure 5:**
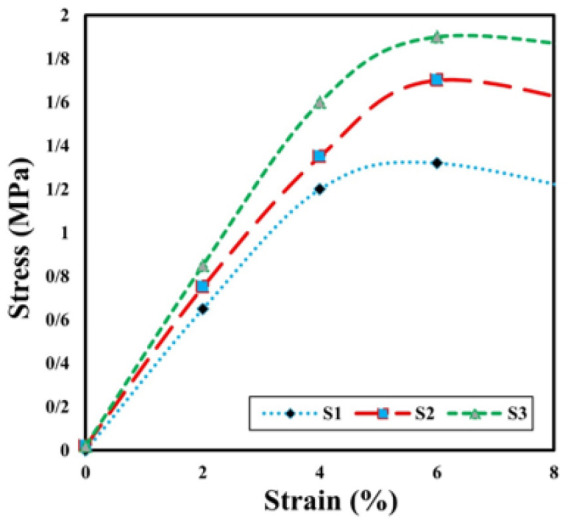
Stress-strain curve of dry state bio-nanocomposite scaffolds using load and displacement data

**Figure 6:**
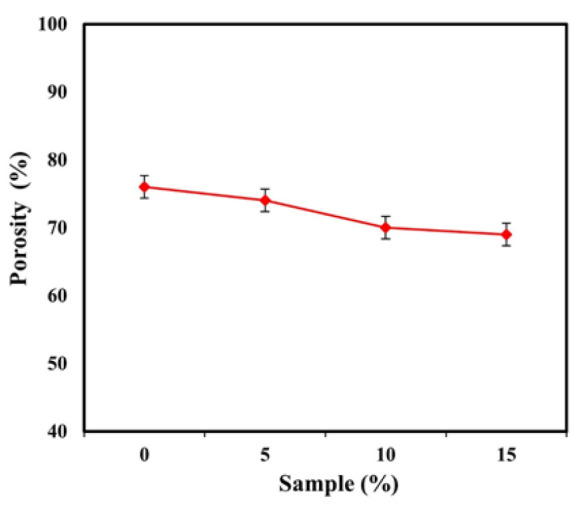
Porosity percentage of bio-nanocomposite scaffolds with 0–15 wt% ZnO-NP, determined using Archimedes principle

ZnO-NP: Zinc oxide nanoparticles

**Figure 7:**
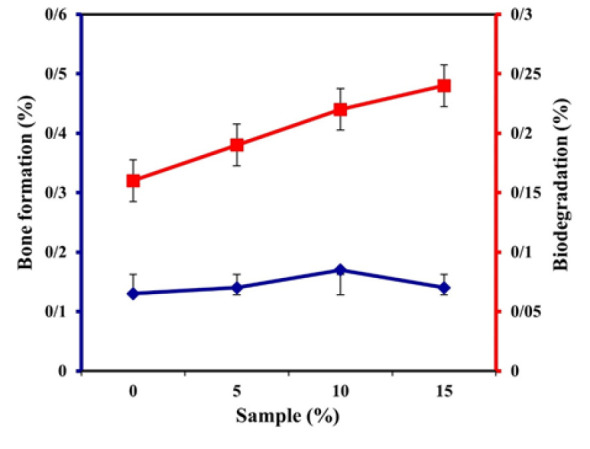
Formation and degradation (%) of apatite within the result of weight changes for porous scaffolds of bio-nanocomposites in SBF and PBS, respectively

SBF: Simulated body fluid; PBS: Phosphate-buffered saline

**Table 2:** Size and porosity results (%) obtained with J-Image and the Archimedes principle for the prepared bio-nanocomposite scaffolds

**Antibacterial (%)**	**Ultimate stress (MPa)**	**Crystalline size (nm)**	**Porosity** **(%)**	**Pore size** **(micron)**	**Scaffold’s identity**
**0.16**	0.71	90	85±5	50-100	S1
**0.17**	1.75	110	79±5	60-111	S2
**0.22**	4.36	132	75±5	65-120	S3
**0.25**	4.38	145	73±5	70-130	S4

**Figure 8:**
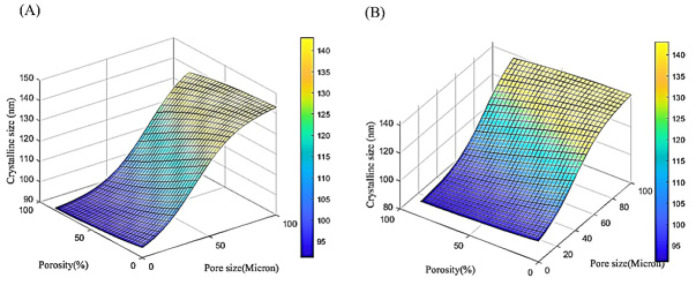
ANN prediction of crystal size (in nanometers) based on the experimental conditions

ANN: Artificial neural network

**Figure 9:**
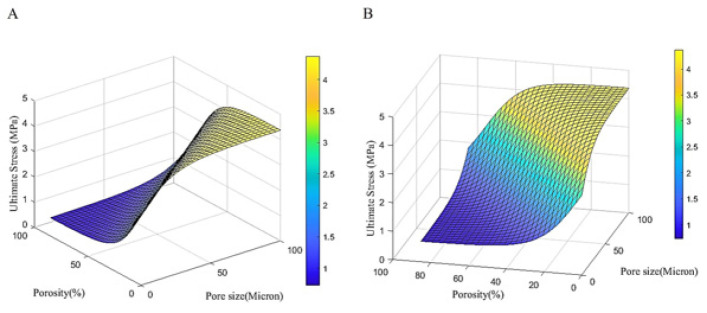
ANN prediction of ultimate stress (in MPa) based on the experimental conditions

ANN: Artificial neural network

**Figure 10:**
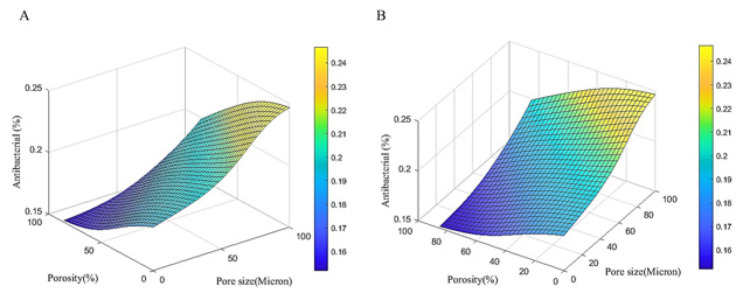
ANN prediction of antibacterial activity (as a percentage) based on the experimental conditions

**Figure 11:**
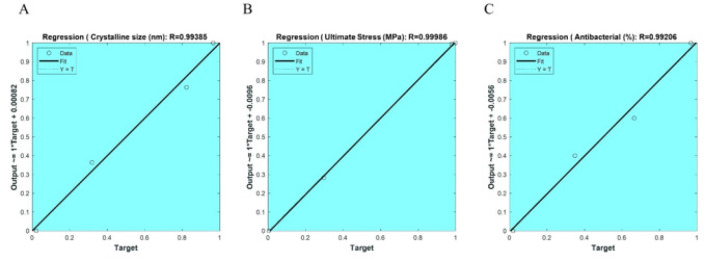
Linear regression plots demonstrating the error of ANN in predicting crystal size (nm), ultimate stress (MPa), and antibacterial activity (%)

The results of the linear regression analysis presented in Figure 11 confirm that ANN was able to predict the crystal size, ultimate stress, and antibacterial activity with very high accuracy, with an error of less than 1% compared to the target values in Table 2.

ANN: Artificial neural network

ANN: Artificial neural network

**Figure 12:**
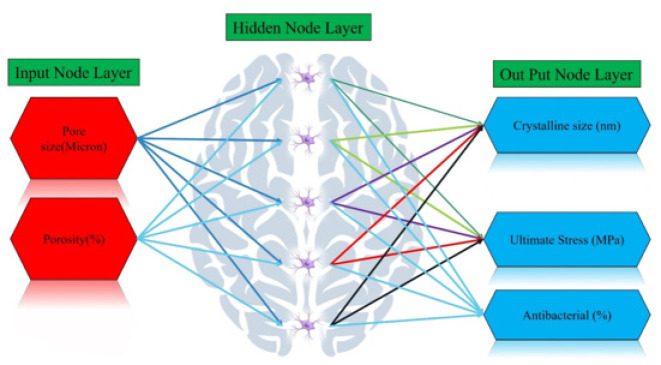
Schematic representation of the ANN architecture, consisting of one hidden layer with 5 neurons and 2 input variables (pore size in microns and porosity percentage), used to predict crystal size (nm), ultimate stress (MPa), and antibacterial activity (%) for the 4 experimental samples

ANN: Artificial neural network

## Results

Liao *et al*. (16) used sodium alginate and chitosan to improve the HA properties in bone tissue and prepared the composite by *in-situ* synthesis method. Microscopic photographs showed that Na-Alg crystals were uniformly distributed throughout the polymer matrix and that the bone strength was appropriately produced. This composite was tested for suitable biodegradability in SBF, and favorable results were obtained. Studies in implants and prosthetics have shown that combating bacterial growth is a decisive factor in the reconstruction of bone tissue (17). One of the best ways to prevent infection is to build material in the implant that can kill the bacteria that cause the infection (18). The sample’s pH and compatibility study with the blood showed that adding 0.1% by weight of ZnO-NP to the scaffold increases its tensile strength, and this bio-nanocomposite has a high potential for use in bone tissue engineering. XRD, SEM, FTIR, and tensile strength test was used to measure tensile strength, maximum length increases, and minimum reduction in sample area and yield stress, and finally, biological activity and bio-compatibility, pH changes, and the swelling behavior of the scaffolds after immersion were determined in SBF, prepared by the Kokubo method, and SC was determined (24). The scaffold apatite capacity was also determined using the SBF solution for 28 days. Figure 1 shows the schematic illustration of the design process for the bio-nanocomposite disc produced using antibacterial agents and sodium alginate. 

***XRD analysis***

The XRD spectra of the porous scaffolds in pure and composite structures are shown in Figure 2. Figure 2 shows pure sodium alginate XRD, HA, and HA/sodium alginate bio-nanocomposite samples. The commercial HA nanoparticles Na-Alg as a matrix were introduced as a result of an effective method for the synthesis of bio-nanocomposites. Since alginates are unparalleled in nature, wider peaks are found to be 23.15 degrees for alginates, while peaks at 32.20° to dispersion (300) are related to HA crystalline nanoparticles (27). This confirms the successful synthesis of bio-nanocomposite HA/Na-Alg powder. Apparently, after the synthesis of the bio-nanocomposite, there was a clear change and decrease in the crystallinity of the main peaks of pure crystalline HA and the biopolymer alginate. This indicates the presence of a bond between the biopolymer matrix and the HA powder. A crystalline HA nanoparticle precipitated in bio-nanocomposites, peak at 32.2 (300) is gradually weakened.

***Fourier transform infrared spectroscopy (FTIR)***

Figure 3 shows the FTIR spectroscopy performed to describe composite functional groups. Typically, FTIR is used to further assess surface chemistry and material properties. In the FTIR spectrum, a number of specific bands of Alg in 1033 cm^-1^ (tensile C-O), 1458 cm^-1^ (ethyl -CH_2_), 1769 cm^-1^ (tensile carbonyl), 2851 cm^-1^ (-CH tensile), and 3566 cm^-1^ (-OH tensile) are clearly seen. 

*Scanning electron microscopy (SEM)*

The SEM images show well-established interconnections between porosity, which play an important role in feeding cellular nutrition, oxygen release, blood vessels, growth factor, and waste disposal from cellular cells in any composite (28). As the literature has introduced, the ideal microparticle size depends on different cells and tissues geometry and shape (25-32). They have different dimensions, and the ideal pore size should be at least a few times larger than the cell size, nearly 20-40 microns, as shown in Figure 4 (a-c).

The polymer forms a thin wall with attached ZnO-NP bioceramic, showing a smaller density than HA particles. Also, according to SEM images, the size of the cavities was reduced by adding ZnO-NP to the scaffold’s bio-nanocomposite. According to SEM images in Figure 4 (a-c) obtained from the S1, S2, and S3 samples, the cavities found in the S3 scaffolds have a more spherical microstructure than the S2 scaffolds, and the relationship between the porous cross-sectional area and their interconnectedness is clearly visible. From the SEM images, it was determined that by increasing the amount of ZnO-NP and the intermixture of the walls, the ZnO-NP were found to be white particles on the surface of the samples. The porous solids microstructure has very high levels of strength in the case of the sample with 10–15 wt% ZnO-NP and very tiny pore volumes that increase absorption capacity. Therefore, the methods of drug delivery in the porous solids are very much considered. With the addition of ZnO-NPs, the number of nanosized particles in the scaffold increases; on the other hand, the accumulation and nanoparticle clotting also increase. In general, due to the high level of HA and ZnO-NPs, it is impossible to completely prevent the accumulation of nanoparticles. By using a standard mixer device, the particles were minimized to the extent possible. The number of HA particles with the dimensions of 50–100 nm was more than that of pumping and accumulated particles with a size greater than 100 nm. The properties of porous bio-nanocomposite scaffolds depend on the size of the based NPs in the composite. In such a way, composite scaffolds contain a small number of smaller particles in comparison with composite scaffolds with identical particles in larger dimensions and have better efficiency and mechanical performances. 

***Mechanical properties***

The tensile test result for the four specimens shows the elastic modulus and maximum strength of the bio-nanocomposite scaffolds in the stress-strain curves, as shown in Figure 5. Sample 3 encountered low mechanical strength and was destroyed at the beginning of loading. As can be seen, the elastic modulus of scaffolds increases more than four times with increasing ZnO-NP in samples 1 and 2 from 0.071 MPa to 1.75 MPa. It is expected that sample 4, with the highest amount of ZnO-NP additive, has the highest modulus of elasticity.

Also, sample 3 had the highest tensile strength and the highest ceramic phase value compared to sample 1. Accordingly, the mechanical performance of the standard sample of the bio-nanocomposite scaffolds is particularly satisfactory, and the mechanical values ​​obtained are in agreement with the reported range for trabecular bone (29-35).

The porosity evaluation using Archimedes and Image-J software was evaluated, and the results are presented in Figure 6. The SEM image of the scaffold for various samples with different ZnO-NP is shown. The holes’ structure is honeycomb and interconnected. The number and size of porosity are very important parameters in determining the mechanical properties and scaffolds’ biocompatibility. The porosity and average size of the cavity of the bio-nanocomposite scaffold are given in Table 2. As shown in Figure 8 (c), all samples have porous structures and related holes. These pores interact with each other, and as a result, there is the possibility of transferring food and oxygen to the growth of cells. In order to measure the porosity diameter, six cavities from each scaffold were measured. With respect to the average cavities, the size of the cavities is smaller by increasing ZnO-NP. The presence of such a phenomenon is due to the higher concentrations in the ceramic phase-containing samples, which is an effective barrier to prevent liquid from passing through the freeze-drying process and creating a slope in pore size.

***Bioactivity evaluation (apatite formation)***

The samples were immersed in SBF for 28 days to analyze the calcium phosphate behavior in the sodium alginate corresponding to various amounts of ZnO-NP. The concentration of calcium ion increases after exposure to the SBF sample for 3 and 7 days. Figure 7 shows the percentages of apatite formation and degradation as a result of weight changes for scaffolds. The moisture and microstructural stability regarding the basic elements of the tissue and scaffolds play an essential role in tissue engineering for nutrition and cell growth. Inflation studies on current bio-nanocomposite scaffolds show a very high swelling rate along proper porosity ratio. 

## Discussion

Recent literature has proposed novel approaches for bone regeneration and osteoporosis management (25-28). A single-stage, customized reconstruction method was suggested utilizing variations of deep iliac circumflex artery perforator chimeric flaps for combined bone and soft tissue defects (29-37). Another study showed that targeting loop 3 of sclerostin maintains its cardiovascular protective function while also promoting bone formation (38-42). The effects of pulsed electromagnetic fields on genes related to osteoblast and osteoclast development were investigated in a postmenopausal osteoporosis mouse model (43-47). Biomaterials were rationally engineered for bone hemostasis and defect repair (48-50). Advances in 3D printed polymer scaffolds for bone were summarized, and a magnetically-driven mini robot was created using asymmetric laser-induced graphene. Recent studies have evaluated various materials and technologies for craniofacial and dental regeneration (50). A study (51) showed a narrative review on the use of animal models in craniofacial regenerative medicine. Researchers (52) discussed the cross-talk between cells and current bioceramics in bone regeneration. Tebyaniyan *et al*. (53) presented an overview of current antibacterial agents in dental bonding systems. Other researchers (54) discussed current infections of the orofacial region regarding treatment, diagnosis, and epidemiology. Mosaddad *et al*. (55) reviewed green alternatives as antimicrobial agents for mitigating periodontal diseases. Another study (56) showed an updated comprehensive review on dental luting cements. Many studies in the literature evaluated synthetic materials in craniofacial regenerative medicine (57), the role of epigenetics in dental stem cells (58), current natural/chemical materials and technologies for periodontal regeneration (59), current and advanced nanomaterials in dentistry (60), decellularized and biological scaffolds (61), natural bioactive materials for bone and tooth regeneration (62), the role and application of stem cells in dental regeneration (63), biocompatible materials for oral drug delivery (64), and properties of developed scaffolds for bone regeneration (65-68). Glass ionomers are a unique type of dental restorative material that combine the benefits of glass and ionomer cements. These materials are composed of a silicate glass powder and a polyacrylic acid liquid, which react to form a strong, adhesive, and biocompatible material (69-74). According to Figure 7, which shows the behavior of the bio-nanocomposite scaffolds of both SBF and SC solutions, the presence and increase of ZnO-NP in the bio-nanocomposite scaffold increased the water absorption of the samples. The increase in ZnO-NP results in the production of chemical bonds in polymeric structure, which can reduce the attraction of the bio-nanocomposite and thus reduce the percentage of inflation. As seen from the behavior of the bio-nanocomposite scaffolds, water molecules first penetrated into the scaffold pores and within the structure of the scaffold, and the water absorption rate increased for all samples. However, the osmotic pressure difference between the solution and the scaffold may be reduced by increasing the water scaffolding interactions, and after several hours, the inflation rate is reduced. The results of the antibacterial test indicate that *Staphylococcus aureus* bacteria have a lower resistance to the *E. coli* bacteria than the HA/Na-Alg composite scaffold with ZnO. HA composite scaffold in the culture medium of *E. coli* and *S. aureus* did not produce a growth hole, but the growth halo around the HA/Na-Alg/ZnO composite scaffold in the medium of bacteria, *E. coli*, and *S. aureus* clearly can be seen. The diameter of the growth halo in the medium of *E. coli* and *S. aureus* was 15 and 30 microns, respectively. 

The current study employed a feedforward neural network to predict the changes in crystal size (in nanometers), ultimate stress (in megapascals), and antibacterial activity (as a percentage) as a function of increasing pore size (0 to 100 microns) and porosity percentage (0 to 85%), as outlined in Table 2. The predicted behavioral trends were investigated and are presented in Figure 8. 

Figure 8 shows that increasing the porosity percentage decreases the crystal size, while increasing pore size leads to an increase in crystal size, indicating a direct relationship between pore size and crystal size. Figure 9 demonstrates that increasing porosity percentage decreases the ultimate stress, but increasing pore size leads to an increase in ultimate stress, suggesting a direct relationship between pore size and ultimate stress. 

Figure 10 shows that, similar to the previous 2 cases, increasing the porosity percentage decreases the antibacterial activity, while increasing the pore size enhances the antibacterial percentage, indicating a direct relationship between pore size and antibacterial activity. 

The key results are that the crystal size is maximized when the pore size is at its maximum and the porosity percentage is less than 50%, the ultimate stress is maximized when the pore size is at its maximum and the porosity percentage is less than 20%, and the antibacterial activity is highest when the porosity percentage is lowest and the pore size is highest. These observations demonstrate the direct relationships between pore size and the studied material properties. Figure 12 shows the schematic representation of the ANN architecture employed in this study. The ANN model consists of a single hidden layer with 5 neurons, and 2 input variables: pore size (in microns) and percentage of porosity. 

This ANN architecture was used to predict three key material properties for the 4 experimental samples: crystal size (nanometers), ultimate stress (MPa), and antibacterial activity (expressed as a percentage). The inclusion of the pore size and porosity percentage as input variables suggests that the researchers aimed to investigate how these structural characteristics of the material influenced the final crystal size, mechanical strength, and antibacterial performance. By using an ANN approach, the study sought to develop a predictive model that could estimate these important material attributes based on the provided input parameters.

## Conclusion

This study presented a comprehensive strategy to improve osteoporosis management in patients with fragility fractures. The proposed multi-modal intervention aims to address key gaps in current post-fracture care through precise fracture risk assessment, patient-tailored treatment decisions, adherence support, and coordinated care across healthcare providers. If proven effective through a randomized controlled pilot trial, this coordinated, risk-stratified approach could significantly improve health outcomes for many patients suffering from osteoporosis worldwide. Additionally, the study developed novel ZnO-reinforced sodium alginate- HA scaffolds for bone tissue engineering applications. A variety of characterization techniques demonstrated that incorporating ZnO nanoparticles enhanced the mechanical properties, bioactivity, porosity, and antibacterial activity of the scaffolds. Furthermore, an ANN model accurately predicted how changes in pore size and porosity percentage impacted other material attributes. This predictive modeling approach can aid the design of scaffolds for optimal bone regeneration. In conclusion, this research proposed innovative solutions to two major healthcare issues - optimizing osteoporosis management after fractures and advancing hard tissue repair with antibacterial scaffolds. Rigorously evaluating the interventions through clinical trials and modeling scaffold properties with neural networks can help validate and refine the approaches. Positive results from such evaluations may ultimately lead to improved patient outcomes and regenerative therapies for damaged bone and skin tissues.

## Data Availability

The datasets supporting the conclusions of this study are included in the article.

## References

[1] Salerno A, Oliviero M, Di Maio E, Netti PA, Rofani C, Colosimo A (2010). Design of novel three-phase PCL/TZ–HA biomaterials for use in bone regeneration applications. J Mater Sci Mater Med.

[2] Costa-Pinto AR, Correlo VM, Sol PC, Bhattacharya M, Charbord P, Delorme B (2009). Osteogenic differentiation of human bone marrow mesenchymal stem cells seeded on melt based chitosan scaffolds for bone tissue engineering applications. Biomacromolecules.

[3] Butscher A, Bohner M, Hofmann S, Gauckler L, Müller R (2011). Structural and material approaches to bone tissue engineering in powder-based three-dimensional printing. Acta Biomater.

[4] Zhang L, Webster TJ (2009). Nanotechnology and nanomaterials: promises for improved tissue regeneration. Nano Today.

[5] Fatalla AA, Arzani S, Veseli E, Khademi A, Khandan A, Fahmy MD (2023). Revolutionizing systematic reviews and meta-analyses: the role of artificial intelligence in evidence synthesis. Dental Hypotheses.

[6] Khandan A, Khosravi M, Roustazadeh D, Aghadavoudi F (2024). Impact of alumina and carbon nanotubes on mechanical properties of a composite: molecular dynamic (MD) simulation. Iran J Chemist Chem Eng.

[7] Moarrefzadeh A, Morovvati M R, Angili S N, Smaisim G F, Khandan A, Toghraie D (2023). Fabrication and finite element simulation of 3D printed poly L-lactic acid scaffolds coated with alginate/carbon nanotubes for bone engineering applications. Int J Biol Macromol.

[8] Lee M, Dunn J C, Wu B M (2005). Scaffold fabrication by indirect three-dimensional printing. Biomaterials.

[9] Heydary H A, Karamian E, Poorazizi E, Khandan A, Heydaripour J (2015). A novel nano-fiber of Iranian gum tragacanth-polyvinyl alcohol/nanoclay composite for wound healing applications. Proced Mater Sci.

[10] Karamian E, Motamedi M R K, Khandan A, Soltani P, Maghsoudi S (2014). An in vitro evaluation of novel NHA/zircon plasma coating on 316L stainless steel dental implant. Prog Nat Sci Mater Int.

[11] Rahman F F, Bonfield W, Cameron R, Patel M P, Braden M, Pearson G (2004). Water uptake of poly (ethylmethacrylate)/tetrahydrofurfuryl methacrylate polymer systems modified with tricalcium phosphate and hydroxyapatite. Key Eng Mater.

[12] Wang L, Shelton R M, Cooper P R, Lawson M, Triffitt J T, Barralet J E (2003). Evaluation of sodium alginate for bone marrow cell tissue engineering. Biomaterials.

[13] Kanasan N, Adzila S, Mustaffa N A, Sidi S M, Rahman M N A, Nasir N F (2017). The effects of sintering temperature on densification and mechanical properties of hydroxyapatite/sodium alginate biocomposites. MATEC Web Conf.

[14] Rajkumar M, Meenakshisundaram N, Rajendran V (2011). Development of nanocomposites based on hydroxyapatite/sodium alginate: synthesis and characterisation. Mater Charact.

[15] Kanasan N, Adzila S, AzimahMustaffa N, Gurubaran P (2017). The effect of sodium alginate on the properties of hydroxyapatite. Procedia Eng.

[16] Liao J, Li Y, Li H, Liu J, Xie Y, Wang J (2018). Preparation, bioactivity and mechanism of nano-hydroxyapatite/sodium alginate/chitosan bone repair material. J Appl Biomater Funct Mater.

[17] Safaei M M, Abedinzadeh R, Khandan A, Barbaz-Isfahani R, Toghraie D (2023). Synergistic effect of graphene nanosheets and copper oxide nanoparticles on mechanical and thermal properties of composites: experimental and simulation investigations. Mater Sci Eng.

[18] Andrade FAC (2013). Desenvolvimento de hidroxiapatita contendo nanopartículas de prata com propriedades antibacterianas.

[19] Chen W, Liu Y, Courtney H S, Bettenga M, Agrawal C M (2016). In vitro anti-bacterial and biological properties of magnetron co-sputtered silver-containing hydroxyapatite coating. Biomaterials.

[20] Salmani M M, Hashemian M, Khandan A (2020). Therapeutic effect of magnetic nanoparticles on calcium silicate bioceramic in alternating field for biomedical application. Ceram Int.

[21] Mohandas A, Sudheesh Kumar P T, Raja B, Lakshmanan V K, Jayakumar R (2015). Exploration of alginate hydrogel/nano zinc oxide composite bandages for infected wounds. Int J Nanomed.

[22] Khandan A, Jazayeri H, Fahmy M D, Razavi M (2017). Hydrogels: types, structure, properties, and applications. Biomat Tiss Eng.

[23] Zhang R, Ma P X (1999). Poly (α‐hydroxyl acids)/hydroxyapatite porous composites for bone‐tissue engineering I Preparation and morphology. J Biomed Mater.

[24] Kokubo T, Takadama H (2006). How useful is SBF in predicting in vivo bone bioactivity?. Biomaterials.

[25] Khandan A, Esmaeili S (2019). Fabrication of polycaprolactone and polylactic acid shapeless scaffolds via fused deposition modelling technology. J Adv Mater Process.

[26] Rajaei A, Kazemian M, Khandan A (2022). Investigation of mechanical stability of lithium disilicate ceramic reinforced with titanium nanoparticles. Nanomed Res J.

[27] Zadeh Dadashi M, Kazemian M, Malekipour Esfahani M (2023). Color match of porcelain veneer light-cure resin cements with their respective try-in pastes: chemical stability. Nanochemist Res.

[28] Heydary H A, Karamian E, Poorazizi E, Heydaripour J, Khandan A (2015). Electrospun of polymer/bioceramic nanocomposite as a new soft tissue for biomedical applications. J Asian Ceram Soc.

[29] Lee S, Porter M, Wasko S, Lau G, Chen P Y, Novitskaya E E (2012). Potential bone replacement materials prepared by two methods. MRS-OPL.

[30] Khandan A, Karamian E, Bonakdarchian M (2014). Mechanochemical synthesis evaluation of nanocrystalline bone-derived bioceramic powder using for bone tissue engineering. Dent Hypotheses.

[31] Ghayour H, Abdellahi M, Ozada N, Jabbrzare S, Khandan A (2017). Hyperthermia application of zinc doped nickel ferrite nanoparticles. J Phys Chem Solids.

[32] Khandan A, Ozada N (2017). Bredigite-magnetite (Ca7MgSi4O16-Fe3O4) nanoparticles: A study on their magnetic properties. J Alloys Compd.

[34] Kazemi A, Abdellahi M, Khajeh-Sharafabadi A, Khandan A, Ozada N (2017). Study of in vitro bioactivity and mechanical properties of diopside nano-bioceramic synthesized by a facile method using eggshell as raw material. Mater Sci Eng.

[35] Sharafabadi A K, Abdellahi M, Kazemi A, Khandan A, Ozada N (2017). A novel and economical route for synthesizing akermanite (Ca2MgSi2O7) nano-bioceramic. Mater Sci Eng.

[36] Karamian E, Abdellahi M, Khandan A, Abdellah S (2016). Introducing the fluorine doped natural hydroxyapatite-titania nanobiocomposite ceramic. J Alloys Compd.

[37] Khandan A, Abdellahi M, Barenji R V, Ozada N, Karamian E (2015). Introducing natural hydroxyapatite-diopside (NHA-Di) nano-bioceramic coating. Ceram Int.

[38] Chen S, Li Y, Zhi S, Ding Z, Huang Y, Wang W (2020). lncRNA xist regulates osteoblast differentiation by sponging miR-19a-3p in aging-induced osteoporosis. Aging Dis.

[39] Zhang Y, Qing L, Luo G, Ahmadpoor X, Li X, Wu P (2023). Variations in deep iliac circumflex artery perforator chimeric flap design for single-stage customized-reconstruction of composite bone and soft-tissue defect. J Plast Reconstr Aesthet Surg.

[40] Yu Y, Wang L, Ni S, Li D, Liu J, Chu H Y (2022). Targeting loop3 of sclerostin preserves its cardiovascular protective action and promotes bone formation. Nat Commun.

[41] Song Z H, Xie W, Zhu S Y, Pan J J, Zhou L Y, He C Q (2018). Effects of PEMFs on Osx, Ocn, TRAP, and CTSK gene expression in postmenopausal osteoporosis model mice. Int J Clin Exp Pathol.

[42] Zhang Y, Zhao C, Zhang H, Chen M, Meng Y, Pan Y (2023). Association between serum soluble α-klotho and bone mineral density (BMD) in middle-aged and older adults in the United States: a population-based cross-sectional study. Aging Clin Exp Res.

[43] Gai Y, Yin Y, Guan L, Zhang S, Chen J, Yang J (2023). Rational design of bioactive materials for bone hemostasis and defect repair. Cyborg Bionic Syst.

[44] Bahadori S, Williams J M, Collard S, Swain I (2023). Can a purposeful walk intervention with a distance goal using an activity monitor improve individuals’ daily activity and function post total hip replacement surgery A randomized pilot trial. Cyborg Bionic Syst.

[45] Jiang N, Zhang J, Li Z, Zhu S S (2023). Scaffold-based tissue engineering strategies for temporomandibular joint disc regeneration and replacement. Eur Cells Mater.

[46] Xu Y, Zhang F, Zhai W, Cheng S, Li J, Wang Y (2022). Unraveling of advances in 3D-printed polymer-based bone scaffolds. Polymers.

[47] Chen Y, Guo Y, Xie B, Jin F, Ma L, Zhang H (2024). Lightweight and drift-free magnetically actuated millirobots via asymmetric laser-induced graphene. Nat Commun.

[48] Zhong Y, Li J, Luo J, Chen F (2021). Advances in bone turnover markers (PINP and CTX) in optimizing anti-resorptive and anabolic therapies against osteoporosis. Discov Med.

[49] Kabeer A S, Osmani H T, Patel J, Robinson P, Ahmed N (2023). The adult with low back pain: causes, diagnosis, imaging features and management. Br J Hosp Med.

[50] Zheng J, Yue R, Yang R, Wu Q, Wu Y, Huang M (2022). Visualization of zika virus infection via a light-initiated bio-orthogonal cycloaddition labeling strategy. Front Bioeng Biotechnol.

[51] Mosaddad S A, Hussain A, Tebyaniyan H (2024). Exploring the use of animal models in craniofacial regenerative medicine: a narrative review. Tissue Eng Part B Rev.

[52] Khayatan D, Bagherzadeh Oskouei A, Alam M, Mohammadikhah M, Badkoobeh A, Golkar M (2024). Cross talk between cells and the current bioceramics in bone regeneration: a comprehensive review. Cell Transplant.

[53] Tebyaniyan H, Hussain A, Vivian M (2023). Current antibacterial agents in dental bonding systems: a comprehensive overview. Future Microbiol.

[54] Tahmasebi E, Keshvad A, Alam M, Abbasi K, Rahimi S, Nouri F (2023). Current infections of the orofacial region: treatment, diagnosis, and epidemiology. Life.

[55] Mosaddad S A, Hussain A, Tebyaniyan H (2023). Green alternatives as antimicrobial agents in mitigating periodontal diseases: a narrative review. Microorganisms.

[56] Heboyan A, Vardanyan A, Karobari M I, Marya A, Avagyan T, Tebyaniyan H (2023). Dental luting cements: an updated comprehensive review. Molecules.

[57] Yazdanian M, Alam M, Abbasi K, Rahbar M, Farjood A, Tahmasebi E (2022). Synthetic materials in craniofacial regenerative medicine: a comprehensive overview. Front Bioeng Biotechnol.

[58] Hussain A, Tebyaniyan H, Khayatan D (2022). The role of epigenetic in dental and oral regenerative medicine by different types of dental stem cells: a comprehensive overview. Stem Cells Int.

[59] Barzegar P E F, Ranjbar R, Yazdanian M, Tahmasebi E, Alam M, Abbasi K (2022). The current natural/chemical materials and innovative technologies in periodontal diseases therapy and regeneration: A narrative review. Mater Today Commun.

[60] Yazdanian M, Rahmani A, Tahmasebi E, Tebyanian H, Yazdanian A, Mosaddad S A (2021). Current and advanced nanomaterials in dentistry as regeneration agents: an update. Mini Rev Med Chem.

[61] Yazdanian M, Arefi A H, Alam M, Abbasi K, Tebyaniyan H, Tahmasebi E (2021). Decellularized and biological scaffolds in dental and craniofacial tissue engineering: a comprehensive overview. J Mater Res Technol.

[62] Moghadam ET, Yazdanian M, Alam M, Tebyanian H, Tafazoli A, Tahmasebi E (2021). Current natural bioactive materials in bone and tooth regeneration in dentistry: a comprehensive overview. J Mater Res Technol.

[63] Soudi A, Yazdanian M, Ranjbar R, Tebyanian H, Yazdanian A, Tahmasebi E (2021). Role and application of stem cells in dental regeneration: a comprehensive overview. EXCLI J.

[64] Hakim L K, Yazdanian M, Alam M, Abbasi K, Tebyaniyan H, Tahmasebi E (2021). Biocompatible and biomaterials application in drug delivery system in oral cavity. Evid Based Complement Alternat Med.

[65] Yazdanian M, Tabesh H, Houshmand B, Tebyanian H, Soufdoost R S, Tahmasebi E (2020). Fabrication and properties of βTCP/Zeolite/Gelatin scaffold as developed scaffold in bone regeneration: in vitro and in vivo studies. Biocybern Biomed Eng.

[66] Tahmasebi E, Alam M, Yazdanian M, Tebyanian H, Yazdanian A, Seifalian A (2020). Current biocompatible materials in oral regeneration: a comprehensive overview of composite materials. J Mater Res Technol.

[67] Mosaddad S A, Yazdanian M, Tebyanian H, Tahmasebi E, Yazdanian A, Seifalian A (2020). Fabrication and properties of developed collagen/strontium-doped bioglass scaffolds for bone tissue engineering. J Mater Res Technol.

[68] Soufdoost R S, Yazdanian M, Tahmasebi E, Yazdanian A, Tebyanian H, Karami A (2019). In vitro and in vivo evaluation of novel tadalafil/β-TCP/collagen scaffold for bone regeneration: a rabbit critical-size calvarial defect study. Biocybern Biomed Eng.

[69] Hussein H A M, Al-Judy H J (2023). Effect of incorporation of boron nitride nanoparticles on impact strength and surface roughness of heat cure poly methyl methacrylate resin: an in vitro study. Dent Hypotheses.

[70] Kamarian S, Barbaz-Isfahan R (2024). Optimal flammability and thermal buckling resistance of eco-friendly abaca fiber/polypropylene/egg shell powder/halloysite nanotubes composites. Adv Nano Res.

[71] Alimirzaei S, Barbaz-Isfahani R, Khodaei A, Najafabadi M A, Sadighi M (2024). Investigating the flexural behavior of nanomodified multi-delaminated composites using acoustic emission technique. Ultrasonics.

[72] Kamarian S, Bodaghi M, Isfahani R B, Song J I (2022). A comparison between the effects of shape memory alloys and carbon nanotubes on the thermal buckling of laminated composite beams. Mech Based Des Struct Mach.

[73] Turki O H, Jafar Z J (2023). Antibacterial activity of Juglans regia L dry husk extract against Streptococcus mutans and Lactobacillus: an in vitro study. Dent Hypotheses.

[74] Hasan Z R, Al-Hasani N R, Mahmood M A, Ibrahim A I (2023). Effect of amoxicillin and azithromycin suspensions on microhardness of sliver reinforced and nano resin-modified glass ionomers: an in vitro study. Dent Hypotheses.

